# Diagnosis of Tooth Prognosis Using Artificial Intelligence

**DOI:** 10.3390/diagnostics12061422

**Published:** 2022-06-09

**Authors:** Sang J. Lee, Dahee Chung, Akiko Asano, Daisuke Sasaki, Masahiko Maeno, Yoshiki Ishida, Takuya Kobayashi, Yukinori Kuwajima, John D. Da Silva, Shigemi Nagai

**Affiliations:** 1 Department of Restorative Dentistry and Biomaterial Sciences, Harvard School of Dental Medicine, Boston, MA 02115, USA; sang_lee@hsdm.harvard.edu (S.J.L.); john_dasilva@hsdm.harvard.edu (J.D.D.S.); 2 Harvard School of Dental Medicine, Boston, MA 02115, USA; dahee_chung@hsdm.harvard.edu; 3 Department of Restorative Dentistry, School of Dental Medicine, Iwate Medical University, Morioka 020-8505, Japan; akiasano@iwate-med.ac.jp; 4 Department of Periodontology, School of Dental Medicine, Iwate Medical University, Morioka 020-8505, Japan; daisukes@iwate-med.ac.jp; 5 Department of Adhesive Dentistry, School of Life Dentistry at Tokyo, The Nippon Dental University, Chiyoda-ku, Tokyo 102-8159, Japan; mmaeno@tky.ndu.ac.jp; 6 Department of Dental Materials Science, School of Life Dentistry at Tokyo, The Nippon Dental University, Chiyoda-ku, Tokyo 102-8159, Japan; yishida@tky.ndu.ac.jp; 7 Department of Oral Rehabilitation, School of Dental Medicine, Iwate Medical University, Morioka 020-8505, Japan; kobataku@iwate-med.ac.jp; 8 Department of Orthodontics, School of Dental Medicine, Iwate Medical University, Morioka 020-8505, Japan; ykuwaji@iwate-med.ac.jp; 9 Department of Oral Medicine, Infection and Immunity, Harvard School of Dental Medicine, Boston, MA 02115, USA

**Keywords:** diagnosis, tooth prognosis, artificial intelligence (AI), machine learning, treatment plan, prosthodontics

## Abstract

The accurate diagnosis of individual tooth prognosis has to be determined comprehensively in consideration of the broader treatment plan. The objective of this study was to establish an effective artificial intelligence (AI)-based module for an accurate tooth prognosis decision based on the Harvard School of Dental Medicine (HSDM) comprehensive treatment planning curriculum (CTPC). The tooth prognosis of 2359 teeth from 94 cases was evaluated with 1 to 5 levels (1—Hopeless, 5—Good condition for long term) by two groups (Model-A with 16, and Model-B with 13 examiners) based on 17 clinical determining factors selected from the HSDM-CTPC. Three AI machine-learning methods including gradient boosting classifier, decision tree classifier, and random forest classifier were used to create an algorithm. These three methods were evaluated against the gold standard data determined by consensus of three experienced prosthodontists, and their accuracy was analyzed. The decision tree classifier indicated the highest accuracy at 0.8413 (Model-A) and 0.7523 (Model-B). Accuracy with the gradient boosting classifier and the random forest classifier was 0.6896, 0.6687, and 0.8413, 0.7523, respectively. Overall, the decision tree classifier had the best accuracy among the three methods. The study contributes to the implementation of AI in the decision-making process of tooth prognosis in consideration of the treatment plan.

## 1. Introduction

Treatment planning in dentistry serves to address a patient’s chief complaint and reestablish the patient’s oral health. A treatment plan should be based on an accurate diagnosis and evaluation of the prognosis. Prognosis is determined by comprehensive clinical knowledge, experience, and patient-specific factors. Moreover, in complex cases, treatment planning is predicated on multidisciplinary analysis and requires the evaluation of not only the individual tooth, but also the entire oral structure, which involves the plane of occlusion, the vertical dimension of occlusion, and the arch form. Thus, treatment planning remains one of the most challenging aspects of clinical practice and dental education.

Many classification systems have been developed to determine the tooth prognosis from each discipline [1,2,3,4,5,6,7,8,9]; however, there is a lack of an integrated and comprehensive system based on an evidence-based multidisciplinary standard. Furthermore, the existing prognostic systems are based on empirical situations, which often leads to different outcomes in complex cases.

Artificial intelligence (AI) has been recently applied in medicine to provide algorithms for making clinical decisions [10,11,12,13,14]. AI is a term that is used for the study, development, and investigation of any computer systems that exhibit “intelligent behavior”, and machine learning is a special branch of AI where the system learns specific statistical patterns in a given dataset to predict the behavior of new data samples [15,16]. Machine learning specifically provides methods suited for complex prediction tasks by training the algorithms to recognize and capture statistical patterns in a given dataset. Therefore, the goal is to identify similar patterns in new test data with high precision and use them for various applications [17].

AI has been used in dentistry to make the process of diagnosis more accurate and efficient in various dental specialties. These AI models have been used in the detection and diagnosis of dental caries, vertical root fractures, apical lesions, salivary gland diseases, maxillary sinusitis, osteoporosis, cancerous lesions, alveolar bone loss, predicting orthodontic extractions, cephalometric analysis, and age and gender determination [18,19,20,21]. For instance, machine-learning algorithms and computer automation provided considerably good performance in detecting dental caries in periapical radiographs, according to Lee et al. [22,23,24,25]. Studies [26,27,28,29] also utilized machine-learning methods to find the most significant factors predicting implant systems and prognosis. It is also reported that dentists have become dependent on computer applications to gain insights for clinical decision making [30,31,32]. Machine-learning algorithms were utilized to identify predictors for tooth loss from the National Health and Nutrition Examination survey and assisted clinicians in prioritizing interventions to prevent tooth loss [33]. However, to date, little is known about AI implemented in comprehensive dental treatment planning.

The Harvard School of Dental Medicine (HSDM) implemented a comprehensive multidisciplinary education in 1994, and a comprehensive treatment planning curriculum (CTPC) has since been conducted. Case presentation seminars to discuss multidisciplinary comprehensive treatment plans have been performed both in predoctoral and advanced graduation education. The CTPC teaches multiple options of treatment plans. Firstly, an ideal plan should be determined based on accurate clinical diagnosis, then alternative plans and limited plans are created considering individual limitations, such as finances, treatment duration, and medical history. Comprehensive treatment plans are approved by senior educators in consultation with faculty specialists. During this process, input from the standpoint of prosthodontics are the most critical factors to determine long-term tooth prognosis, and the prosthodontic faculty play a significant role in this curriculum.

The ultimate goal of this project is to develop an integrated and comprehensive system to create a treatment plan by AI-based machine-learning algorithms. The specific aim of this study was to create an effective AI-based module that allows for accurate clinical decisions on tooth prognosis, taking the ideal treatment plan into account. Furthermore, this study compared the prediction accuracy of algorithms between two learning datasets, with or without the involvement of an experienced prosthodontist’s judgment.

## 2. Materials and Methods

This study was approved by the Institutional Review Board (IRB) at Harvard Medical School and clinical cases of 94 patients with 2359 teeth were used in this study. The access to the electric record in axiUm (Exan, Vancouver, BC, Canada) of these cases and case presentation documents were obtained.

### 2.1. Determination of Clinical Parameters

**Figure 1:**
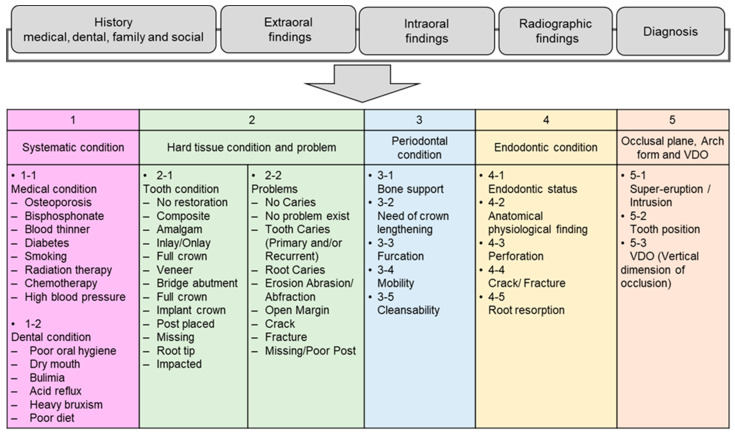
Seventeen key parameters used for determination of tooth prognosis.

The clinical faculty from the disciplines of prosthodontics, periodontics, general dentistry, endodontics, orthodontics, and oral surgery (20 faculty in total, Table 1) reviewed the outlined items of HSDM-CTPC; medical history, dental history, family history, social history, extraoral examination findings, intraoral examination findings, radiographic findings, occlusal examination findings, and diagnosis. Through multiple discussions among the faculty members, 17 key parameters including medical and dental conditions, hard tissue, and periodontal, endodontic, prosthodontic, and orthodontic conditions were determined for a training dataset for machine learning (Figure 1). These parameters were selected based on the weight on tooth prognosis for an ideal treatment plan to be considered. Then, data on each parameter were entered into an Excel sheet (Microsoft 365, Washington, DC, USA) using electronic records in axiUm and case presentation documents.

### 2.2. Ranking of Tooth Prognosis

**Figure 2:**
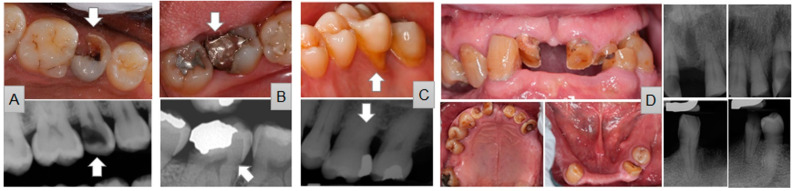
Samples of teeth for ranking. (**A**): #4 with extensive caries. (**B**): #31 with tooth fracture and open margin. (**C**): #14 with significant bone loss. (**D**): #7, 8, 9 10, 11 and #21 with loss of occlusion.

Sixteen examiners (Table 2, Model-A) individually graded the tooth prognosis of 2359 teeth by ranking from 1 to 5, with 1: hopeless tooth (signifying severe problems requiring tooth extraction) and 5: healthy tooth (signifying good conditions for long-term). The examiners graded the tooth prognosis in consideration of future comprehensive treatment plans. In addition to the dataset in the Excel sheet, clinical pictures (static and excursions) and full-mouth radiography (FMX) images of each case were also provided (Figure 2). Model-B (Table 2, Model-B) was set by excluding three experienced prosthodontic faculty who participated in the creation of a gold standard. These three HSDM prosthodontic faculty, who had played a significant role in HSDM clinical education and patient care, individually ranked the tooth prognosis first as an examiner of Model-A. Approximately 3 months later, they determined a gold standard of prognosis of 2359 teeth based on their consensus by discussion (Table 2, gold standard) without knowing their original rank.

### 2.3. Analysis of AI Machine Learning Models

This study then devised the data preprocessing pipeline that cleaned up the data in the Excel sheet into vectors and was used for training and testing the AI models. Using this preprocessing pipeline, this study trained three AI machine-learning methods: (1) a gradient boosting classifier, (2) a decision tree classifier, and (3) a random forest classifier. These three methods were tested against the gold standard data and the performance of each method was evaluated in terms of accuracy.

(1) The gradient boosting classifier is a generalization of boosting to arbitrary differentiable loss functions. A gradient boosting decision tree is an accurate and effective procedure that can be used for both regression and classification problems in a variety of areas. This method helps to reduce errors by decreasing bias.
(2) The decision tree classifier is a non-parametric supervised learning method used for classification and regression. The method helps to create a model that predicts the value of a target variable by learning simple decision rules inferred from the data features. A tree can be seen as a piecewise constant approximation.
(3) The random forest classifier is a non-parametric supervised learning method used for classification and regression.

The goal was to create a model that predicts the value of a target variable by learning simple decision rules inferred from the data features. A tree represents a piecewise constant approximation, and it is a commonly used algorithm for simple measurement for the prediction factors.

## 3. Results

### 3.1. Statistics of Tooth Prognosis Rank

**Figure 3:**
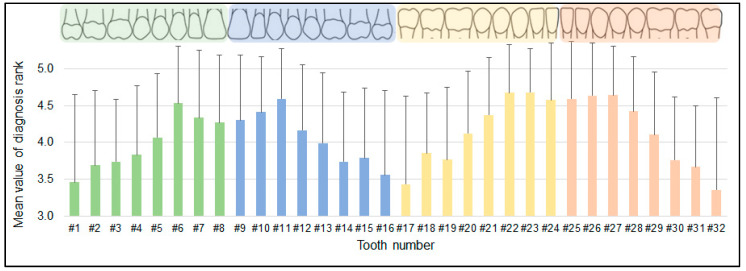
Mean value of the prognosis rank on each tooth type. The specific pattern of diagnosis rank was observed.

The mean prognosis rank among 32 tooth types was 4.19 ± 0.93, max: 4.68, min: 3.43 (Figure 3). The specific patterns of prognosis rank were observed among the tooth types. One-way analysis of variance (ANOVA) indicated a significant difference in tooth prognosis rank among the tooth types (*p* < 0.01). Tukey’s post hoc test showed that the mean values of the prognosis rank of canines, central incisors, and lateral incisors were significantly higher than for premolars and molars (*p* < 0.01, Table 3).

**Figure 4:**
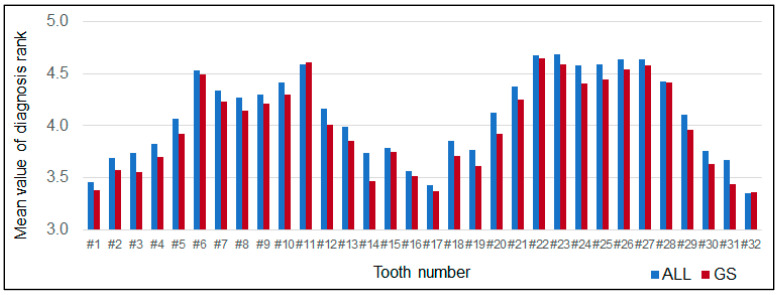
Comparison of the mean value of the diagnosis rank on each tooth between the learning data and gold standard rank (GS). The mean value of the learning data (4.19 ± 0.93) was significantly higher than the GS (4.07 ± 0.98, *p* < 0.0001, unpaired *t* test).

**Figure 5:**
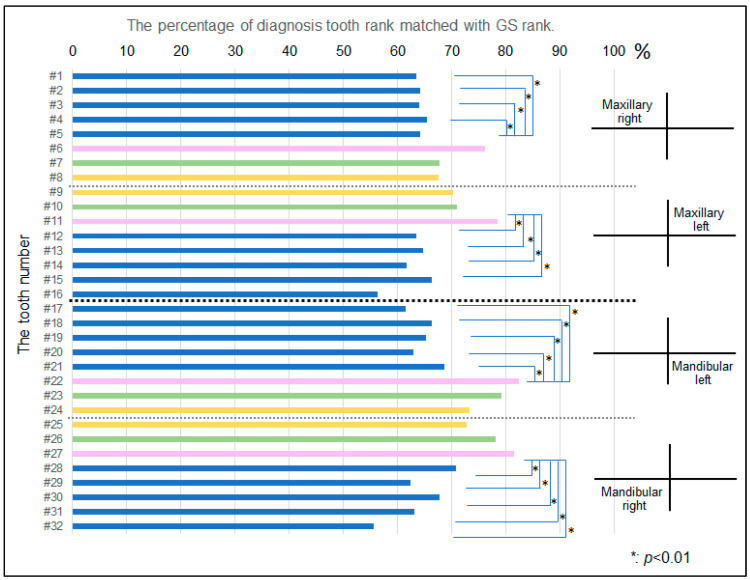
Percentage of diagnosis tooth rank matched with GS rank. The matching percentage was significantly different among the type of tooth (<0.01, one-way ANOVA), and canines (#6, 7, 11, 22, and 27) indicated a significantly higher matching rate than premolars and molars (*p* < 0.01). Blue: premolars and molars, Pink: Canined; Green: Lateral incisors; Yellow: Central Incisors.

**Figure 6:**
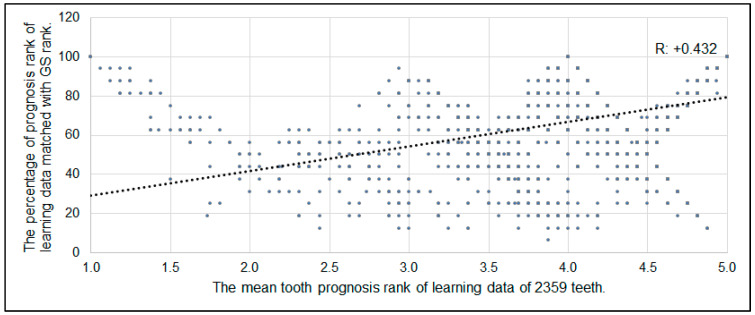
Association between diagnosis tooth rank and matching percentage with GS rank. The Pearson’s correlation indicated that there was a significant medium positive relationship (*p* < 0.001) between the percentage of prognosis rank of learning data matched with GS rank and the mean tooth prognosis rank of learning data of 2359 teeth.

The mean value of the gold standard of the prognosis rank of all 2359 teeth was lower than the rank of learning data (*p* < 0.001, Figure 4). The percentage of the prognosis rank of the learning data matched with GS rank indicated a particular trend among the tooth types (Figure 5). Among 2359 teeth ranked by 17 examiners, 69.24% of teeth matched with the GS rank. The matching percentage was significantly different among the type of tooth (<0.01, one-way ANOVA). The canines indicated a significantly higher matching rate than premolars and molars (*p* < 0.01). There was an association between the prognosis rank and its matching rate with GS. The Pearson’s correlation coefficient was +0.432 (*p* < 0.001, Figure 6).

### 3.2. Accuracy of AI Machine Learning Methods

The accuracy of the performance of the three machine learning methods for the two models is shown in Table 4. Using the gradient boosting classifier, Model-A achieved an accuracy of 0.6896, while Model-B achieved an accuracy of 0.6687. With the decision tree classifier, Model-A achieved an accuracy of 0.8413, while Model-B achieved an accuracy of 0.7523. Using the random forest classifier, Model-A achieved an accuracy of 0.8312 and Model-B achieved an accuracy of 0.7421. Overall, the decision tree classifier method had the best accuracy among the three methods. Model-A indicated a higher accuracy than Model-B for all three machine-learning methods.

## 4. Discussion

The three machine-learning algorithm methods used in this study come with their individual set of advantages. A random forest classifier was used because, according to Uddin et al., it demonstrated superior accuracy among other supervised machine-learning algorithms including artificial neural networks, k-nearest neighbor, logistic regression, decision tree, naïve Bayes, and support vector machine. It generates a large number of decision trees based on random subsamples of the training set while randomly varying the features used in the trees, which allows for the modeling of nonlinear effects [34]. A decision tree classifier was utilized for this study because it demonstrated superior results in studies that used clinical and demographic data [34]. The decision tree classifier iteratively subdivides the training set by selecting feature cutoffs so it can model nonlinear effects, and is easily interpretable as long as the tree depth is low. Since the use of different datasets, different preprocessing steps, and different performance metrics may lead to different conclusions, three different methods were used to compare and analyze what method works best for our dataset. Among the three machine-learning algorithm methods, the decision tree classifier had the best accuracy of 84.13%.

The use of electronic dental records has been increasing and clinical data have been digitalized, which allows for the use of AI to interpret the data. According to Umer et al., modern medicine brought a revolution with the application of AI in clinical decision-making processes [35]. AI and machine-learning algorithms incorporate a computational model that utilizes and processes information on many datasets with critical parameters to predict an outcome. The procedure relies on the recognition of patterns using training data and applies this knowledge to the prediction of outcomes in a different dataset or test data. In dentistry, there are vast quantities of different types of data, including restorative and periodontal charts, the results of diagnostic and laboratory tests, radiographs, and extraoral and intraoral images [36]. These datasets can be transcribed into machine-learning models to generate outputs such as diagnosis, treatment recommendations, and future disease predictions [36]. While scientific publications related to AI in healthcare have quadrupled in the past decade [37], dental literature on the subject of AI has not been as common. In a recent systemic review, only 43 original research articles on AI were identified in peer-reviewed journals over past 12 years [18]. Therefore, this study contributes to the implementation of AI in the decision-making process for accurate tooth prognosis in consideration of the treatment plan, and sets the stage for future applications of machine learning in the dental field.

This study utilized data from a multidisciplinary study team comprised of leading specialists from prosthodontics, periodontics, endodontics, and long-term experienced clinician educators at HSDM. Since various factors can impact tooth prognosis, this integration of different disciplines is critical and meaningful to achieve better predictions. Model-A showed a higher accuracy than Model-B in all three machine-learning methods. As Model-A includes the datasets trained by a group including experienced prosthodontists, the accuracy turned out to be higher and more predictable in consensus with the gold standard. This result is comparable to the findings that the accuracy of machine learning-generated analytics and predictive modeling is highly dependent on the type and quality of the data from which the machine learning system is learning [36]. Thus, it is critical to obtain data from experienced clinician educators to build this machine-learning model and the gold standard is applied to the dataset for an accurate and consistent outcome.

This is the first attempt to utilize machine-learning algorithms to predict tooth prognosis based on multidisciplinary factors in consideration of the treatment plan. The AI model in the study can improve the access to and quality of dental care and can be used to further develop computational models that serve as the foundation of transformative discoveries by improving the efficiency and safety of dental care through multiple disciplines. The developed models will continuously synthesize their predictive outcome measures and improve their accuracy, allowing clinicians to decide their treatment prognosis in various and complex cases. In the future, real-time online clinical decision support tools can be created by incorporating the machine-learning algorithms developed from this study to facilitate precision medicine in oral care. According to Hung et al., these algorithms can be used as a screening tool in general medical practices, dental clinics, and social service centers or placed online, providing recommendations for dental examinations for those identified as high risk [38].

This study was not without limitations. To evaluate the performance of a machine-learning model, it should be trained on unseen data. To achieve this, this study created the entire dataset from electronic health records and compared various models. This approach might incorporate bias, especially if the dataset is limited and varied. To mitigate this bias, a large dataset is required, which can be achieved by collecting more cases including complex and advanced clinical cases. Another limitation of this study is the use of a solitary performance metric, which has inherent limitations. Therefore, the reporting of more robust performance metrics such as F1 scores, receiver operating curve, and area under the receiver operating curve can be more valid and accurate.

In the future, it is possible to utilize this AI-based machine-learning algorithm to formulate various treatment plan options with determining factors identified in categories such as fixed prosthesis, removable prosthesis, and with or without orthodontics. Furthermore, this AI-based machine-learning algorithm can find modifications of the tooth prognosis decision algorithms with different health policy systems, such as private dental insurance, universal healthcare insurance, and community health-based insurance, as tooth prognosis predication can be variable depending on health policies.

## 5. Conclusions

Within the limitations of the study, it was concluded that an AI-based machine-learning algorithm will be a helpful tool to determine tooth prognosis in consideration of the treatment plan. The comprehensive treatment plan needs to be well considered to diagnose tooth prognosis for long-term oral health and function.

## Figures and Tables

**Table 1:** Discipline distribution of participants for clinical parameter determination (mean ± SD).

	Prosthodontist	Periodontist	Endodontist	Orthodontist	Oral Surgery	General Dentist
Parameter determination	5 faculty	4 faculty	3 faculty	2 faculty	1 faculty	5 faculty
Years of experience	27.4 ± 10.1	16.5 ± 8.2	25.0 ± 7.1	17.5 ± 3.5	15	17.4 ± 7.8

**Table 2:** Distribution of participants for ranking of tooth prognosis (mean ± SD).

	Prosthodontist	Periodontist	General Dentist	Predoc DMD4
Model-A (Years of experience)	6 faculty (26.0 ± 10.5)	3 faculty (15.7 ± 5.5)	3 faculty (7.7 ± 8.1)	4 students (2)
Model-B (Years of experience)	3 faculty (20.0 ± 10.4)	3 faculty (15.7 ± 5.5)	3 faculty (7.7 ± 8.1)	4 students (2)
Gold standard (Years of experience)	3 faculty (32.0 ± 7.9)	-	-	-

**Table 3:** Results of Tukey’s post hoc test. Anterior teeth (#6, 7, 8, 9, 10, 11, 22, 23, 24, 25, 26, and 27) indicated a significantly higher rank than premolars and molars.

	#1	#2	#3	#4	#5		#12	#13	#14	#15	#16
#6	0.0000	0.0000	0.0000	0.0000	0.0025	#9			0.0016	0.0046	0.0031
#7	0.0007	0.0003	0.0016	0.0086		#10			0.0001	0.0002	0.0003
#8	0.0025	0.0019	0.0081	0.0388		#11	0.0406	0.0004	0.0000	0.0000	0.0000
	#17	#18	#19	#20			#25	#26	#27	#28	
#20	0.0018					#25	0.0026	0.0000	0.0000	0.0000	
#21	0.0000	0.0016	0.0003			#26	0.0006	0.0000	0.0000	0.0000	
#22	0.0000	0.0000	0.0000	0.0003		#27	0.0004	0.0000	0.0000	0.0000	
#23	0.0000	0.0000	0.0000	0.0002		#28		0.0001	0.0000	0.0000	
#24	0.0000	0.0000	0.0000	0.0067		#29			0.0204	0.0002	

**Table 4:** Accuracy achieved with different machine-learning methods.

Machine Learning	Model-A			Model-B		
	Accuracy	Mean	SD	Accuracy	Mean	SD
Gradient boosting classifier	0.6896	4.3163	0.8344	0.6687	4.3473	0.8035
Decision tree classifier	0.8413	4.198	0.9307	0.7523	4.265	0.8965
Random forest classifier	0.8312	4.2145	0.9263	0.7421	4.2828	0.8867

## Data Availability

The data presented in this study are available on request from the corresponding author. The data are not publicly available due to privacy restrictions.
